# Expedition to the missing link: Long noncoding RNAs in cardiovascular diseases

**DOI:** 10.1186/s12929-020-00647-w

**Published:** 2020-04-02

**Authors:** Chih-Fan Yeh, Yu-Chen Eugene Chang, Cheng-Yuan Lu, Chin-Feng Hsuan, Wei-Tien Chang, Kai-Chien Yang

**Affiliations:** 1 Graduate Institute and Department of Pharmacology, National Taiwan University School of Medicine, No.1, Sec. 1, Ren-Ai Rd, 1150R Taipei, Taiwan; 2 Division of Cardiology, Department of Internal Medicine, National Taiwan University Hospital, No.1, Sec. 1, Ren-Ai Rd, 1150R Taipei, Taiwan; 3 Division of Cardiology, Department of Internal Medicine, E-Da Dachang Hospital, Kaohsiung, Taiwan; 4 Department of Medicine, I-Shou University School of Medicine, Kaohsiung, Taiwan; 5 Department of Emergency Medicine, National Taiwan University Hospital, Taipei, Taiwan

**Keywords:** Long noncoding RNA, Cardiovascular, Disease, Development

## Abstract

With the advances in deep sequencing-based transcriptome profiling technology, it is now known that human genome is transcribed more pervasively than previously thought. Up to 90% of the human DNA is transcribed, and a large proportion of the human genome is transcribed as long noncoding RNAs (lncRNAs), a heterogenous group of non-coding transcripts longer than 200 nucleotides. Emerging evidence suggests that lncRNAs are functional and contribute to the complex regulatory networks involved in cardiovascular development and diseases. In this article, we will review recent evidence on the roles of lncRNAs in the biological processes of cardiovascular development and disorders. The potential applications of lncRNAs as biomarkers and targets for therapeutics are also discussed.

## Introduction

Transcriptional profiling has been utilized extensively to understand complex disease pathways [1], to identify novel biomarkers for diagnosing and prognosing diseases [2], and to examine the efficacy of therapeutic treatments with cardiovascular diseases [3–5]. Although intriguing results have been generated, previous transcriptional profiling studies in cardiovascular system targeted largely the expression of messenger RNAs (mRNAs) and microRNAs (miRNAs), which in combination account for only ~ 1% of all transcribed RNA species [6]. It is now known that the transcription of the eukaryotic genome is much more pervasive and complex than previously appreciated [6]. It is estimated that up to 90% of the mammalian genome is transcribed [6], and a large proportion of the mammalian genome is transcribed as long non-coding RNAs (lncRNAs), a heterogeneous group of non-coding transcripts longer than 200 nucleotides, encoded from genomic loci within or between (long intergenic RNA, lincRNA) coding genes [7, 8]. LncRNAs have been shown to be functional and involved in specific physiological and pathological processes, including chromatin modification [9, 10], cellular responses to DNA damage [11], stem cell pluripotency/differentiation [12], cell cycle control [13], as well as in the pathogenesis of neurologic diseases [14, 15] and human cancers [16–18]. Functionally, lncRNAs are known for their roles as modulators of transcription, including epigenetic regulation of chromatin structure [19]. In addition, lncRNAs have been shown to function as regulators of post-transcriptional mechanisms including transcript splicing [20], mRNA decay [21], and protein translation [22]. In this regard lncRNAs are unique, functioning not dependent solely on sequence (as with miRNAs) or structure (as for RNA-binding proteins). Rather, lncRNAs seem to function both by sequence homology/complementarity with other nucleic acids, as well as by structure, forming scaffolds for the assembly of macromolecular complexes that regulate biological processes [23]. In this review, we describe and summarize recent evidence on the contributions of lncRNAs to the development of cardiovascular diseases including heart failure (and its precursors like cardiac hypertrophy, myocardial infarction and cardiac fibrosis), atherosclerosis, and arrhythmias. The roles of lncRNAs in cardiac development and their utilization as biomarkers for diagnosing and prognosing cardiovascular diseases are also discussed.

## LncRNAs in the failing heart

The roles of lncRNAs in heart failure (HF) have been investigated extensively using high-throughput RNA sequencing both in human [24–26] and animal models [27–30]. Using deep RNA sequencing on myocardial RNA samples from human HF patients before and after left ventricular assisted device (LVAD) support, we have revealed the dynamic regulation of myocardial transcriptome, including mRNA, miRNA and lncRNA, in advanced HF and after LVAD support. We have also shown that the expression profiles of lncRNA, but not miRNA or mRNAs, can discriminate failing hearts of ischemic vs non-ischemic origins [24], suggesting the importance of lncRNAs in the pathogenesis of HF and reverse remodeling observed with mechanical support.

### LncRNAs and cardiac hypertrophy

Cardiac hypertrophy, caused by abnormal hemodynamic stress or myocardial injury, is initially induced as a compensatory response to maintain cardiac output. This pathological hypertrophy, however, is often accompanied with increased cardiomyocyte death, fetal gene re-expression and interstitial/perivascular fibrosis, and will ultimately progress to cardiac dilatation and heart failure [31]. Recently, Han et al. [32] discovered that a group of lncRNAs derived from the *Myh7* loci, coined myosin heavy chain associated RNA transcripts (*Mhrt*), are protective against cardiac hypertrophy. *Mhrt* is downregulated in the mouse heart with thoracic aortic constriction (TAC)-induced cardiac hypertrophy and was associated with the *Myh6/Myh7* isoform switch, a pathognomonic signature of cardiomyopathy. Conversely, restoring *Mhrt* expression reduced the pathological changes of cardiac hypertrophy. Mechanistically, *Mhrt* was found to function via regulating the Brg1-Hdac-Parp chromatin repressor complex by competitively binding to Brg1, thereby prohibiting it from initiating the *Myh6-*to-*Myh7* switch. This mechanism appears to be conserved in humans, with the finding that human *MHRT* expression is suppressed in myopathic hearts. Later on, Luo et al. [33] uncovered an alternative cardioprotective mechanisms of *Mhrt,* where *Mhrt* reduces cardiac expression of myocardin, a muscle-specific transcription co-activator that promotes the expression of cardiac hypertrophy related genes, by reducing myocardin acetylation via HDAC5. Myocardin also binds to the CarG box of the *Mhrt* promoter and increases the transcriptional activity of *Mhrt*, forming a positive feedback loop. These studies together demonstrate the crucial roles of *Mhrt* in the pathogenesis of cardiac hypertrophy.

Exploiting transcriptome analysis in TAC-induced hypertrophic mouse hearts, Viereck et al. [29] identified a hypertrophy-associated, cardiomyocyte-specific lncRNA *cardiac hypertrophy-associated transcript* (*Chast*). In vitro and in vivo cardiac hypertrophy was observed upon overexpression of *Chast*, whilst attenuation of hypertrophic response was observed when *Chast* was silenced using anti-sense GapmeRs, single-stranded antisense oligonucleotides that allows RNase-H-mediated cleavage of target RNA. The transcription of *Chast* was activated by prohypertrophic nuclear factor of activated T cells (NFAT) signaling. *Chast* downregulates Pleckstrin homology domain-containing protein family M member 1 (*Plekhm1*), a regulator of normal cardiac autophagy, leading to maladaptive cardiac remodeling and hypertrophy. A human analog of *Chast* was also identified and shares similar functions with its mouse counterpart.

Another lncRNA identified via transcriptome analysis on pressure-overload-induced mouse hypertrophic hearts, *cardiac-hypertrophy-associated epigenetic regulator* (*Chaer*), was reported to contribute to cardiac hypertrophy and fibrosis [30]. Mechanistically, *Chaer* interacts with the catalytic subunit of polycomb repressor complex 2 (PRC2) via an EZH2 binding motif, interfering with the targeting and repressive action of PRC2 on the promoter regions of genes involved in cardiac hypertrophy. The mTOR-dependent *Chaer*-PRC2 interaction thus releases pathological gene suppression by H3K27 tri-methylation in cardiomyocytes, resulting in the upregulation of pro-hypertrophic genes including *Anf*, *Myh7*, and *Acta1* in response to hypertrophic stimulation. Furthermore, knockdown of *Chaer* reduced pathologic cardiac remodeling only within a narrow timeframe before TAC surgery, suggesting a critical window immediately following the onset of hypertrophic stress during which epigenetic regulation by the *Chaer*-PRC2 complex occurs. This also suggests an epigenetic checkpoint of PRC2 present in the progression of cardiac hypertrophy.

Recently, studies have shown that one class of lncRNAs modulates physiological and pathological processes through regulation of miRNAs by acting as an endogenous sponge of the target miRNA to prevent miRNAs from reducing the stability or interfering the translation of their target transcripts. These competing endogenous RNAs (ceRNAs) ultimately derepresses its downstream molecular pathways, a mechanism that could either salvage inhibited cellular/physiological processes or reactivate/ magnify responses to pathological stresses.

One of the first ceRNAs to be recognized as a key component in cardiac hypertrophy is *cardiac hypertrophy-related factor* (*CHRF*) [34], which was shown to exert its pro-hypertrophic effects by targeting the anti-hypertrophic miR-489/Myd88 axis. More recently, another study revealed that *CHRF* also regulates the cardiac miR-93/Akt3 axis [35]. In this case, *CHRF* attenuated the repressive effects of miR-93 on Akt3, a protein kinase of the PI3K/Akt signaling pathway critical for the regulation of cardiac contractile function and stress response.

Another ceRNA that has been extensively studied is *myocardial infarction-related transcripts* (*MIAT*). Zhu et al. [36] found that *MIAT* regulates cardiac hypertrophy via sponging miR-150, a miRNA exhibiting anti-hypertrophic effects on cardiomyocyte. P300 was then determined to be the downstream effector of this *MIAT*-miR150 axis in promoting cardiac hypertrophy [37]. Additional studies by Li et al. [38] showed that *MIAT* contributes to cardiac hypertrophy also by influencing the miR-93/TLR4 axis. *MIAT-*mediated sponging of miR-93 led to upregulation of TLR4 and activation of hypertrophic PI3K/Akt/mTOR signaling, thereby leading to AngII-induced cardiac hypertrophy.

Other examples of lncRNAs that regulate cardiac hypertrophy-related miRNA as ceRNAs include *H19* [39], *HOTAIR* [40], *lncRNA-ROR* [41], *MAGI1-IT1* [42], *Meg3* [43], *Plscr4* [44], *SYNE1-AS1* [45], and *XIST* [46, 47]. Table 1 summarizes lncRNAs that are implicated in the pathogenesis of cardiac hypertrophy and heart failure.

**Table 1:** LncRNAs implicated in the pathogenesis of cardiac hypertrophy and heart failure

LncRNA	Target	Physiological/pathological impact	Mechanism involved
Mhrt [32, 33]	Brg1	Protective against pathological cardiac hypertrophy	1. Inhibit Brg1-Hdac-Parp chromatin repressor complex to prohibit initiation of *Myh6-*to-*Myh7* switch 2. Reduce myocardin acetylation/expression via HDAC5
Chast [29]	Plekhm1	Promotes cardiac hypertrophy	Activated by NFAT signaling and downregulates *Plekhm1* to induce cardiac remodeling processes
Chaer [30]	PCR2	Increases pro-hypertrophic gene expression	Interact with PCR2 to disinhibit hypertrophic gene expression
CHRF [34, 35]	1. miR-489 2. miR-93	Induces cardiac hypertrophic responses	1. Sponge miR-489 to increase expression of Myd88 2. Sponge miR-93 to disinhibit PI3K/Akt pathway
MIAT [36–38, 48]	1. miR-150 2. miR-93 3. miR-24	Contributing factor to the pathogenesis of cardiac hypertrophy, myocardial infarction and cardiac fibrosis	1. Sponge miR-150 to increase expression of P300 2. Sponge miR-93 to activate PI3K/Akt/mTOR pathway via TLR4 3. Sponge anti-fibrotic miR-24
HOTAIR [40]	miR-19	Inhibiting progress of cardiac hypertrophy	Sponge miR-19 to derepress PTEN expression
lncRNA-ROR [41]	miR-133	Promoting fetal genes and cardiomyocyte growth	Sponge miR-133
MAGI1-IT1 [42]	miR-302e	Protective against cardiac hypertrophy	Sponge miR-302e to derepress DKK1 and inactivate Wnt/beta-catenin signaling
Meg3 [43]	miR-361-5p	Promotes cardiac hypertrophy	Activated by STAT3 to sponge miR-361-5p and derepress HDAC9
Plscr4 [44]	miR-214	Protective against cardiac hypertrophy	Sponge miR-214 to derepress Mfn2
SYNE1-AS1 [45]	miR-525-5p	Promoting cardiac hypertrophy	Activated by SP1 to sponge miR-525-5p to derepress SP1, forming positive feedback loop
XIST [46, 47]	1. miR-101 2. miR-330-3p	1. Regulating cardiac hypertrophy 2. Attenuating cardiac hypertrophy	1. Sponge miR-101 to derepress TLR2 2. Sponge miR-330-3p to derepress S100B

### LncRNAs and myocardial infarction

Myocardial injury following myocardial infarction (MI) represents a particular challenge in clinical practice as it almost invariably leads to adverse cardiac remodeling and fibrosis, causing ischemic heart failure [49]. Determining the functional contribution of lncRNAs to MI may shed light on previously unknown cellular processes and serve as novel targets for therapeutics.

Studying the effects of lncRNAs on autophagy, Liu et al. [50] found that the lncRNA *cardiac autophagy inhibitory factor* (*CAIF*) showed cardioprotective properties by interacting with p53 protein, thereby blocking it from activating myocardin transcription and subsequent cardiac autophagy.

An interesting alternative mode of action of lncRNA was discovered when Greco et al. [51] studied the effect of β-secretase-1 (BACE1), the enzyme that produces the β-amyloid peptide, and its antisense transcript *BACE1-AS* on HF. They showed that *BACE1-AS* stabilizes the BACE1 sense transcript, resulting in their concordant upregulated expression in cardiomyocytes and endothelial cells. *BACE1-AS* positively regulates the level of BACE1 protein, leading to increased β-amyloid protein, the accumulation of which is toxic to endothelial cells and cardiomyocytes. These data suggest that the *BACE1-AS*/BACE1/β-amyloid pathway contributes to the pathogenesis of HF.

Similar to the case of cardiac hypertrophy, an increasing number of ceRNAs have emerged that regulate numerous processes of myocardial infarction and cardiac infarction through miRNA inhibition.

The ceRNA *cardiac apoptosis-related lncRNA* (*CARL*) [52] was shown to regulate mitochondrial fission and apoptosis by acting as an endogenous sponge of miR-539 in cardiomyocytes, a miRNA that represses the expression of PHB2, a critical inhibitor of mitochondrial fission and apoptosis. Modulating CARL-miR-539 and PHB2 axis thus could be helpful in improving survival of cardiomyocytes and preserving myocardial function.

Another ceRNA *autophagy promoting factor* (*APF*) was reported to target the miR-188-3p/ATG7 axis to induce adaptive cell autophagy in cardiomyocytes after MI [53]. In this study, miR-188-3p was shown to repress ATG7 expression, thereby inhibiting autophagy and causing cell death in cardiomyocytes. *APF* binds with miR-188-3p as a ceRNA and leads to increased ATG7 levels and enhanced cardiac autophagy. Increased cardiac levels of *APF* preserves myocardial function in response to ischemic/reperfusion (I/R) injury.

In addition to cell apoptosis and autophagy, ceRNAs have also been shown to regulate necrosis in cardiomyocytes. *Necrosis-related factor* (*NRF*), for example, regulates cardiomyocyte necrosis by targeting miR-873 and RIPK1 (receptor-interacting serine/threonine-protein kinase 1)/RIPK3 (receptor-interacting serine/threonine-protein kinase 3). In vitro and in vivo experiments reveal that miR-873 inhibits H_2_O_2_-induced necrotic cell death and I/R injury-induced cardiomyocyte necrosis in mouse hearts by targeting RIPK1/RIPK3. NRF, a ceRNA that is transcriptionally activated by p53 upon I/R injury, binds miR-873, leading to increased cardiomyocyte necrosis mediated by RIPK1/RIPK3 [54].

LncRNAs linked to MI are summarized in Table 2.

**Table 2:** LncRNAs linked to myocardial infarction

LncRNA	Target	Physiological/pathological impact	Mechanism involved
CAIF [50]	P53	Preventing detrimental autophagy and cell death of cardiomyocytes	Block p53 from activating myocardin transcription
BACE1-AS [51]	BACE1	Increases expression of toxic β-amyloid protein in endothelial cells and cardiomyocytes	Stabilize β-secretase-1 (BACE1) sense transcript to increase β-amyloid protein production
CARL [52]	miR-539	Regulates mitochondrial fission and apoptosis	Sponge miR-539 to derepress PHB2 expression
APF [53]	miR-188-3p	Induces adaptive cell autophagy after MI	Sponge miR-188-3p to derepress ATG7 expression
NRF [54]	miR-873	Regulates programmed cardiomyocyte necrosis pathways	Activated by p53 to sponge miR-873 and derepress RIPK1/RIPK3 expression

### LncRNAs and cardiac fibrosis

Exploiting the RNA sequencing data obtained from mouse heart following MI, Micheletti et al. [55] identified *Wisp2 super-enhancer-associated RNA* (*WISPER*), a cardiac fibroblast-enriched lncRNA that regulates cardiac fibrosis through association with TIA1-related protein and the regulation of profibrotic lysyl hydroylase 2. Depletion of *WISPER* in vivo repressed fibroblast differentiation and proliferation, inhibiting the development of cardiac fibrosis, suggesting the potential of targeting *WISPER* as an antifibrotic therapeutic strategy.

Maternally expressed gene 3 (*Meg3*), a fibroblast-enriched lncRNA, was reported to be dysregulated in chronic pressure overload-induced cardiac fibrosis. Silencing *Meg3* in cardiac fibroblasts inhibits P53-dependent transcriptional activation of matrix metalloproteinase-2 (MMP-2), an essential mediator of cardiac fibrosis and remodeling. In vivo inhibition of *Meg3* prevented pressure overload-induced MMP-2 induction, leading to reduced cardiac fibrosis and improved diastolic function [56].

LncRNA MALAT1 has been shown to be upregulated in infarction-induced fibrotic mouse heart and angiotensin II (AngII)-treated cardiac fibroblasts, leading to the inhibition and downregulation of miR-145, a negative regulator of of TGFβ1. Knocking down MALAT1 in cardiac fibroblasts restored miR-145 expression and prevented AngII-induced fibroblast profileration, collagen production and α-SMA expression [57].

In addition to its role in MI and cardiac hypertrophy, recent research has shown that lncRNA *MIAT* also play roles in cardiac fibrosis. MIAT is markedly upregulated in post-infarct fibrotic mouse heart, which was accompanied by by downregulation of miR-24 and upregulation of Furin and TGFβ1 [48]. Knocking down MIAT1 in the mouse heart or cardiac fibroblasts attenuated fibrogenic response following pathological stimulations.

LncRNAs shown to contribute to cardiac fibrosis are summarized in Table 3.

**Table 3:** LncRNAs contributing to cardiac fibrosis

LncRNA	Target	Physiological/pathological impact	Mechanism involved
WISPER [55]		Promoting cardiac fibrosis	Promotes differentiation and proliferation of cardiac fibroblasts by interacting with TIA1-related protein and regulation of lysyl hydroylase 2
MEG3 [56]	MMP2	Promoting cardiac fibrosis	Required for TGFβ-mediated, P53-dependent MMP-2 transcriptional activity
MALAT1 [57]	miR-145	Promoting cardiac fibrosis	Promotes fibroblast activity and collagen production by downregulating miR-145, a negative regulator of TGFβ
MIAT1 [48]	miR-24	Promoting cardiac fibrosis	Increases fibrosis by sponging miR-24

## LncRNAs in cardiac arrhythmias and electrical remodeling

Electrical remodeling, a process involving the dysregulation/dysfunction of cardiac ion channels and transporters under pathological conditions, creates a proarrhythmogenic substrate that predisposes to the development of atrial and ventricular arrhythmias. Recently, lncRNAs have been shown to contribute to the development of myocardial electrical remodeling and cardiac arrhythmias.

Downregulation of *TCONS_0007546*, a lincRNA reported to act as a competing endogenous RNA of miR-328, was found to shorten the atrial effective refractory period (AERP) and reduce atrial fibrillation (Afib) inducibility in rabbits as a result of downregulation of CACNA1C protein secondary to increased miR-328 levels [58, 59].

Recently, lncRNA metastasis-associated lung adenocarcinoma transcript 1 (*MALAT1)* was found to mediate the cardioprotective effects of fentanyl against myocardial ischemia-reperfusion injury [60]. Zhu et al. performed further study on the potential interaction between *MALAT1* and ion channels. *MALAT1* was highly upregulated in the cardiomyocytes from rats with post-MI ventricular arrhythmias, and like endometrioid adenocarcinoma, *MALAT1* acts as a sponge for miR-200c in cardiomyocytes. The overexpression of *MALAT1* notably inhibited the level of miR-200c, which was shown to target high-mobility group box 1 (HMGB1), leading to reduced levels of Kv4.2 and Kv4.3 channel, α subunits of the potassium channels that encode cardiac transient outward currents (I_to_) [61]. Taken together, lncRNA *MALAT1* regulates cardiac electrophysiology through miR-200c/HMGB1/Kv channel pathway [62].

LncRNAs were also found to contribute to the pathogenesis of ventricular arrhythmia. LncRNA *Kcna2-AS*, for example, negatively regulates *Kcna2*, which encodes Kv1.2 potassium channel subunit that plays a pivotal role in regulating several physiological functions, including heart rate [63]. Long et al. observed that the *Kcna2-AS* level was significantly upregulated, while the expression of *Kcna2* was downregulated, in rat failing cardiac ventricles. In addition, knocking down *Kcna2* in the heart led to reductions in delayed rectifier potassium current I_Ks_ and prolonged action potential (AP) durations in rats. Therefore, the upregulation of cardiac *Kcna2-AS* downregulates *Kcna2/*Kv1.2, leading to increased susceptibility to ventricular arrhythmias in rats with CHF [63].

Connexins (Cx), known as gap junction proteins, are indispensable for the electrical conduction in the myocardium. Recently, lncRNA *CCRR* (cardiac conduction regulatory RNA) was found to regulate connexin 43 (Cx43), normally localized at the intercalated disc that connects cardiomyocytes, via binding with CIP85 (Cx43- interacting protein of 85 kDa). CIP85 interacts with Cx43 and promotes its endocytosis and degradation. Zhang et al. demonstrated that *CCRR* protected Cx43 from endocytic trafficking through forming the CCRR:CIP85 complex. Therefore, overexpression of *CCRR* increased cardiac Cx43 levels, rescued intercellular conduction block and contractile dysfunction in heart failure (HF) mice [64].

Sarco/endoplasmic reticulum Ca^2+^-ATPase (SERCA) serves as the major Ca^2+^ efflux mechanism in cardiomyocytes and plays an essential role in maintaining sarcolemmal Ca^2+^ homeostasis and excitation-contraction (EC) coupling. During HF, the transcript/protein expression levels and enzyme activity of SERCA2a, the principal SERCA isoform in cardiomyocytes, are significantly reduced, leading to delayed clearance of cytosolic Ca^2+^, elevated diastolic [Ca^2+^], reduced SR Ca^2+^ store and decreased peak Ca^2+^ transient, which can lead to cardiac contractile dysfunction and electrical instability. LncRNA *ZFAS1*, which was upregulated with ischemic HF, was found to represses the expression and availability of SERCA2a, resulting in intracellular Ca^2+^ overload in cardiomyocytes. Mechanistically, ZFAS1 directly binds SERCA2a protein, leading to reduced SERCA2a activity and expression levels. Targeting the functional domain by which *ZFAS1* interacts with SERCA2a with an antisense oligonucleotide fragment (As*ZFAS1*-FD) blocks the deleterious effects of ZFAS1 in the heart [65].

Although lncRNAs, by definition, do not encode proteins, recent studies discovered that some lncRNAs actually have open reading frames (ORFs) and may be translated into functional micropeptides [66–68]. Micropeptides translated from putative long non-coding RNAs have been reported to play critical roles in modulating muscle function. Magny et al. reported a 31 amino-acid micropeptide Sacrolamban (Scl), encoded from the ORF of *pncr003:2 L* (putative noncoding RNA 003 in 2 L), in *Drosophila*. They showed that this arthropod peptide exhibits homologous structure and function with sacrolipin and phospholamban in vertebrate cardiac tissue. Scl peptides is localized to *Drosophila* SR membrane and regulates SERCA activity directly. Alteration of the level of Scl leads to dysregulation in calcium trafficking and irregular cardiac contraction [69]. In mammals, a 34 amino-acid micropeptide DWORF (dwarf open reading frame) encoded from lncRNA *LOC100507537*, was highly enriched in muscle tissue. Nelson et al. showed that DWORF shares the same binding sites with other SERCA binding regulators, such as phospholamban (PLN) in cardiac muscle, sacrolipin (SLN) in atrium, or myoregulin (MLN) in fast skeletal muscle, on the M6 transmembrane alpha-helix of SERCA. DWORF acts as a physiological competitive antagonist that interferes the interaction between PLN, SLN, or MLN with SERCA. In contrast with the inhibitory effects of SERCA activity by PLN, SLN, or MLN, increased DWORF activated the calcium influx induced by SERCA that resulted in decreasing the time of individual contraction-relaxation cycle. Increasing DWORF levels in failing heart relieved the inhibition of SERCA2a by PLN and restored cardiac contractile function [70, 71]. Table 4 summarized the aforementioned lncRNAs shown to regulate cardiac arrhythmias and electrical remodeling.

**Table 4:** LncRNAs reported to regulate cardiac arrhythmias and electrical remodeling

LncRNA	Target	Physiological/pathological impact	Mechanism involved
TCONS_0007546 [58, 59]	miR-328	Reduces atrial fibrillation inducibility	Functions as a sponge of miR-328 to derepress CACNA1C expression
MALAT1 [61, 62]	miR-200c	Reduces Kv4.2 & Kv4.3 expression→ reduced I_to_	Sponging miR-200c to derepress HMGB1 expression
Kcna2-AS [63]	Kcna2	Increases susceptibility to ventricular arrhythmia in failing heart	Reducing Kcna2 → reduced I_Ks_ → AP_s_ prolongation in rat failing heart
CCRR [64]	CIP85	Preserving cardiac Cx43 expression, conduction and contractile function	Forms complex with CIP85 and prevents CIP85-mediated Cx43 endocytosis and degradation
ZFAS1 [65]	SERCA2a	Upregulated in ischemic HF, causing impaired contractile function	Repress SERCA2a expression and availability, leading to intracellular Ca^2+^ overload
Sacrolamban (Scl) [69]	SERCA	Mediating the calcium reuptake in cardiomyocytes of *Drosophila*	
DWORF [70, 71]	PLN, SLN, or MLN	Enhance cardiac contractility	Decrease the time of individual contraction-relaxation cycle

## LncRNAs in atherosclerotic vascular diseases

Atherosclerosis is the underlying cause of cardiovascular diseases including coronary artery disease (CAD), cerebrovascular disease, aortic aneurysm, and peripheral arterial occlusive disease (PAOD), which are the precursors of myocardial infarction, stroke, limb ischemia and sudden cardiac death [72]. Collectively, cardiovascular diseases resulting from atherosclerosis have become the leading cause of death around the world, and the incidence is on the rise as a result of the international epidemic of obesity, type 2 diabetes and aging, all of which are potent risk factors for atherosclerosis [73].

The early events that lead to the development of the atherosclerotic plaque include endothelial activation and recruitment of circulating leukocytes to the affected arterial intima, as well as local deposition of lipids and accumulation of vascular smooth muscle cells (VSMCs) and extracellular matrix proteins [74, 75]. VSMCs and endothelial cells (ECs) play crucial roles in vascular remodeling and atherosclerosis. Dysfunction of the endothelial lining of the arterial vasculature is an important contributor to the pathobiology of atherosclerotic cardiovascular disease. Pathological stresses, such as oxidized low-density lipoprotein (oxLDL) and hyperglycemia, lead to changes in lncRNA expression in human EC [76, 77]. On the other hand, atherogenic cytokines and growth factors transform VSMCs from a contractile, quiescent state to an active, synthetic state with increased VSMC proliferation, migration and extracellular matrix protein deposition. In addition, abnormal lipid metabolism contributes critically to the development of atherosclerotic lesions. Emerging evidence suggests that lncRNAs serve as a key regulators of lipid homeostasis. Here, we focus on atherorelevant lncRNAs that function through regulating ECs, VSMCs and lipid metabolism.

### LncRNAs in endothelial cells (ECs)

H19 is one of the first identified maternally imprinted lncRNAs, and is involved in cell differentiation and growth. H19 is abundantly expressed during embryonic development and subsequently suppressed in adult vasculature [78, 79]. Interestingly, even though its expression was only retained in a few adult tissues, H19 was found to be involved in the dysregulation of ECs during atherosclerosis. Hyperglycemia leads to H19 downregulation in ECs, which is linked to impaired angiogenesis in diabetes through repressing insulin-PI3K-Akt signaling. Both Akt activity in ECs and diabetic wound healing were enhanced by extracellular vesicle-mimetic nanovesicles carrying H19 [80], strongly suggesting the critical role of H19 in the pathogenesis of atherosclerosis.

MALAT1 is a conserved lncRNA ubiquitously expressed in a number of cells and tissues, and can be induced in response to many pathological stimulations including hypoxia, high glucose and oxidative stress. Silencing MALAT1 reduced endothelial cell proliferation by inhibiting cell cycle progression through reducing the S-phase cyclins CCNA2, CCNB1, and CCNB2, while increasing the cell cycle inhibitors P21 and P27kip1 [81]. *Malat1*^*−/−*^ mice showed a delayed vessel extension in the retina revascularization, while pharmacological inhibition of MALAT1 reduced blood flow recovery and capillary density after hindlimb ischemia and ameliorate diabetic retinopathy, such as microvascular leakage, and retinal inflammation [81, 82], suggesting an important role of MALAT1 in promoting endothelial cell proliferation and blood vessel growth. Interestingly, genetic deletion *of Malat1* in mice did not impact the recovery of blood flow and capillary density under non-ischemic conditions [83].

LncRNA *MEG3* is found to regulate both angiogenesis and diabetes-related microvascular dysfunction. It has been shown that *MEG3* affects angiogenesis through Notch signaling. In a stroke model of transient middle cerebral artery occlusion, *MEG3* was downregulated. Silencing of *MEG3* resulted in a proangiogenic effect through upregulation of Notch pathway-related genes in both ischemia brains and endothelial cells, leading to enhanced functional recovery and reducing the focal ischemia volume after a stroke [84]. In addition, *MEG3* was highly increased in senescent HUVECs and human atrial samples, whereas silencing MEG3 prevented aging-mediated suppression of vascular sprouting activity and led to improved blood flow in an ischemic hindlimb mouse model by enhancing regenerative angiogenesis [85]. *Meg3*-KO mice showed increased expression of VEGF pathway genes and increased cortical microvessel density in *Meg3*-null embryos, suggesting the importance of *MEG3* in vascularization and angiogenesis [86]. *MEG3* expression is down-regulated in the retinas of streptozotocin (STZ)-induced diabetic mice, where reduced *MEG3* leads to capillary degeneration and increased microvascular leakage and inflammation. In this context, *MEG3*-mediated endothelial regulation is mediated by the activation of PI3K/Akt signaling [87].

Nexilin F-actin–binding protein (NEXN), also called Nexilin, is an actin-binding protein that regulates cell adhesion and migration. NEXN and its anti-sense lncRNA NEXN antisense RNA 1 (NEXN-AS1), expressed both in humans and mice, are reduced in human atherosclerotic plaques; circulating NEXN is also significantly decreased in patients with CAD, MI or HF. NEXN-AS1 interacts with the chromatin remodeler BAZ1A to increase the transcriptional activity of the 5′ flanking region of the *NEXN* gene. NEXN exerts atheroprotective effects by inhibiting endothelial activation and monocyte recruitment via the TLR4/NF-κB–mediated pathway. In addition to endothelial cells, NEXN-AS1 was also shown to modulate NEXN expression in monocytes and VSMCs. *NEXN*^*+/−*^*ApoE*^*−/−*^mice (*NEXN*^*−/−*^*ApoE*^*−/−*^ mice were embryonically lethal) had substantially increased atherosclerotic burden and thinner cap fibroatheroma than *ApoE*^*−/−*^mice, indicating that NEXN plays a protective role against the development of vulnerable atherosclerotic plaques and atherosclerosis [88].

Hemodynamic disturbed flow pattern makes endothelia atherosusceptible, and hence atherosclerosis preferentially develops at sites of curvature, branching and bifurcation in elastic and muscular arteries. MANTIS, a nuclear-localized lncRNA controlled by the histone demethylase JARID1B, regulates angiogenesis by interacting *in trans* with BRG1 and facilitates the binding of BRG1 and BRG1-stimulating factor BAF155 on angiogenic genes [89]. Of note, the expression of MANTIS in endothelial cells was induced by atheroprotective flow and statin through mechanisms involving epigenetic rearrangements and the transcription factors KLF2 and KLF4. MANTIS, especially its Alu element, limits endothelial ICAM-1 expression by reducing the binding of the BRG1 at the ICAM-1 promoter. Interestingly, statins also mediate their atheroprotective effects, at least partially, through MANTIS. Importantly, the expression of MANTIS in human carotid artery endarterectomy material was lower compared with healthy vessels and this effect was prevented by statin therapy [90].

### LncRNAs in vascular smooth muscle cells

Genome-wide association studies (GWAS) revealed a strong association between DNA sequence variants on chromosome 9p21.3 and the risk of coronary artery disease, accounting for ~ 10–15% of disease in non-African populations [91–94]. The 9p21.3 risk locus is adjacent to the last exons of the antisense ncRNA in the INK4 locus (ANRIL) and encompasses multiple single nucleotide polymorphisms (SNPs). The function of ANRIL remains incompletely understood, but a number of studies suggest ANRIL regulates cell proliferation and senescence of VSMCs either by Alu motif-dependent *trans* regulation of atherorelevant genes through polycomb protein complexes [95], or by regulating miR-181a/*Sirt1* [96]. Using iPSC-derived VSMCs, VSMCs carrying 9p21.3 risk allele exhibit globally altered transcriptional networks that intersect with previously identified coronary artery disease (CAD) risk genes and pathways and risk-dependent gene networks drive cell state instability, partially through ANRIL [97]. Deleting the risk haplotype rescues VSMC stability, while expression ANRIL induces risk phenotypes in non-risk VSMCs. This provides evidence for crosstalk between CAD risk loci and possible vascular therapeutic targets.

Although H19 expression is normally suppressed in adult vasculature, it is re-expressed in the neointimal lesions, particularly in VSMCs, upon vascular injury [79]. H19 is barely detectable in proliferating neointimal VSMCs, but becomes highly abundant in postconfluent, differentiated neointimal VSMCs, suggesting its role in the phenotypic changes of VSMCs during post-injury neointimal formation [79]. H19 is by far the only lncRNA that has been linked to abdominal aortic aneurysm (AAA). H19 was highly upregulated in the medial layer, particularly in VSMCs, of aortic tissues from human AAA patients, as well as from mouse models of AAA induced by Ang II or porcine pancreatic elastase. Increased H19 levels promote VSMC apoptosis, independent of miR-675, through cytoplasmic interaction with hypoxia-inducible factor 1α and sequential p53 stabilization. Knockdown of H19 with site-specific antisense oligonucleotides (LNA-GapmeRs) in vivo significantly limited aneurysm growth in mouse models of AAA [98]. However, opposite effects of H19 have also been reported, where knockdown of H19 using siRNA suppressed proliferation and promoted apoptosis in human aortic VSMCs following the treatment of ox-LDL, both in normoxic and hypoxic condition [99, 100]. The contradictory effects of H19 on VSMCs observed in different studies may result from differences in the knockdown strategy and the atherogenic stimuli used.

LncRNA MALAT1 has also been shown to contribute to vascular diseases. In VSMCs, MALAT1 form a ternary complex with histone deacetylase HDAC9 and the chromatin-remodeling enzyme BRG1 to recruit PRC2 and inhibit gene expression of contractile proteins by epigenetical silencing [101]. Targeting HDAC9-BRG1-MALAT1 complex by deletion of *Malat1* or *Hdac9* in vivo restores contractile protein gene expression, improves aortic mural architecture, and inhibits experimental aneurysm growth in *Fbn1*^*C1039G/+*^ (Marfan syndrome) mouse model.

### LncRNAs in lipid metabolism

LncLSTR (liver-specific triglyceride regulator) is a mouse lncRNA predominantly expressed in liver, but no human orthologue was found. Mice with liver-specific depletion of lncLSTR exhibit a marked reduction in plasma triglyceride (TG) levels through clearance of TG by ApoC2-mediated lipoprotein lipase activation. Interestingly, lncLSTR doesn’t regulate ApoC2 through a cell-autonomous mechanism. It was found lncLSTR regulates expression of Cyp8b1, a rate-limiting enzyme in bile acid biosynthesis, by interacting with RNA-binding protein TDP-43 to reduce TDP-43 occupancy and inhibition of the Cyp8b1 promoter. The change in bile pool composition by Cyp8b1 alters the activity of bile acid receptor FXR in liver, leading to induction of ApoC2 genes, an important component of very low-density lipoproteins and chylomicrons. Moreover, FXR deficiency did not completely block the lipid-lowing effects of lncLSTR depletion, which indicated the interaction between lncLSTR and TDP-43 might have additional molecular and physiological functions [102]. Importantly, lncLSTR not only plays a unique role in TG metabolism, but also exhibits tissue- and species-specific characteristics of lncRNAs.

LeXis (liver-expressed LXR induced sequence) is a conserved, liver-enriched lncRNA that is robustly induced by high cholesterol diet and activation of liver X receptor (LXR). LeXis inhibits cholesterol biosynthesis by acting as an important conduit between the nutrient-sensing nuclear receptor liver X receptor and the master cholesterol regulator SREBP2. LeXis reduces cholesterol synthesis by interacting with and affecting the DNA interactions of RALY, a heterogeneous ribonucleoprotein that acts as a transcriptional cofactor for cholesterol biosynthetic genes in the mouse liver [103]. Notably, a gene therapy utilizing AAV8.hTBG.*LeXis* significantly reduced atherosclerotic burden in *Ldlr*^*−/−*^ mice [104].

MeXis (macrophage-expressed LXR-induced sequence), a conserved and macrophage-enriched lncRNAs, is an amplifier of LXR-dependent transcription of the gene *Abca1*, a critical regulator of cholesterol efflux. MeXis expression is markedly induced by physiologic lipid signals, such as oxidized or acetylated LDL, and LXR activation. *MeXis*^*−/−*^ mice show reduced *Abca1* expression in a tissue-selective manner in response to Western diet, and MeXis is required for maximal *Abca1* expression in the face of macrophage cholesterol loading. MeXis modulates nearby gene *Abca1* expression through interacting with and guiding promoter binding of the transcriptional coactivator DDX17. Furthermore, loss of MeXis in mouse bone marrow cells alters chromosome architecture at the *Abca1* locus, impairs cellular responses to cholesterol overload, and accelerates the development of atherosclerosis, supporting role of macrophage MeXis in *Abca1*-mediated cholesterol efflux and atherosclerosis development [105]. Table 5 summarized lncRNAs that are implicated in the development of atherosclerotic cardiovascular diseases.

**Table 5:** LncRNAs implicated in the development of atherosclerotic cardiovascular diseases

LncRNA	Clinical Relevance	Physiological/pathological impact	Mechanism involved
H19 [79, 98–100]	↑ in VSMC of neointima and AAA	EC: Improved wound healing in diabetic rats VSMC: promotes SMC apoptosis and development of AAA	EC: Hyperglycemia-induced reduction impaired angiogenesis in diabetes through insulin PI3K-Akt pathway VSMC: Regulate VSMC apoptosis through interaction with HIF1α and sequential p53 stabilization
MALAT1 [81–83, 101]	↑ in VSMC and EC in response to stresses like hypoxia or high glucose	EC: Deletion delayed vessel extension in the retina revascularization, and reduced blood flow recovery after hindlimb ischemia VSMC: Deletion restores contractile protein gene expression, improves aortic mural architecture, and inhibits experimental aneurysm growth	EC: Inhibit cell cycle progression through reducing the S-phase cyclins while increasing the cell cycle inhibitory genes P21 and P27kip1
ANRIL [95, 96]	Adjacent to 9p21.3 CAD risk locus	VSMC: Deleting the risk haplotype rescues VSMC stability	VSMC: regulated cell proliferation and senescence of VSMCs either by a scaffold, guiding effector-proteins to chromatin, or by regulating miR-181a/Sirt1
MEG3 [85–87]	Downregulated with stroke and diabetic retina	EC: Deletion results in a proangiogenic effect	EC: Deletion upregulate Notch VEGF pathway-related genes
NEXN-AS1 [88]	Reduced in arterial plaques	NEXN plays a protective role against development of vulnerable atherosclerotic plaques and atherosclerosis	EC: inhibit endothelial activation and monocyte recruitment via the TLR4/NF-κB–mediated pathway
MANTIS [89, 90]	Induced by disturbed flow; Reduced in arterial plaques	EC: statins mediate their positive effects through the MANTIS	EC: flow sensitive, limit endothelial ICAM-1 expression by reducing the binding of the BRG1 at the ICAM-1 promoter
LncLSTR [102]		LncLSTR deletion reduces plasma triglyceride levels by increasing hepatic expression of lipoprotein ApoC2	Regulate expression of Cyp8b1, which alters the activity of bile acid receptor FXR in liver, leading to induction of ApoC2 genes
LeXis [103, 104]		Reduce hepatic cholesterol biosynthesis, serum cholesterol and atherosclerotic lesion	Inhibit cholesterol biosynthesis as a conduit between LXR and SREBP2
MeXis [105]		Deletion in mouse bone marrow cells impairs cellular responses to cholesterol overload, and accelerates atherosclerosis	Modulate nearby gene Abca1 expression through interacting with and guiding promoter binding of the transcriptional coactivator DDX17

## LncRNAs in cardiac development

LncRNA *Bvht* (Braveheart), transcribed from a regulatory locus located on mouse chromosome 18, has specific expression pattern during the mouse embryonic stem cells (ESC) differentiation and cardiac lineage commitment. The depletion of *Bvht* using short hairpin RNA (shRNA) results in failure to promote a MESP1-driven gene expression program because *Bvht* regulates upstream of *Mesp1*. *Bvht* has also been revealed to react to SUZ12, a core component of PRC2, a complex that has histone methyltransferase activity and mediates transcriptional repression. Therefore, *Bvht* is thought to play a critical role in mediating gene expression in the developing heart [106]. *Linc1405* (Large intergenic non-coding RNAs-1405) was also shown to be involved in regulation of *Mesp1* expression. Eomes, a transcription factor participating in the control of developmental progression, was recruited to *Mesp1* promotor by *Linc1405*. Meanwhile, it has been shown that the Linc1405/Eomes complex united WDR5 and GCN5 to activate *Mesp1* in primitive streak, thereby promoting cardiac mesoderm specification and cardiogenesis [107].

Like *Bvht*, *CARMEN (Cardiac mesoderm enhancer-associated noncoding RNA)*, a lncRNA overlapping with the miR143/miR145 locus, also interacts with EZH2 and SUZ12 of the PRC2 complex. *CARMEN* knockdown leads to inhibition of cardiac specification and differentiation in cardiac precursor cells. *CARMEN* silencing also represses *Bvht* induction without affecting miR143/miR145 expression, indicating that *CARMEN* controls upstream of *Bvht.* Moreover, *CARMEN* also regulates the expression of cardiac transcription factors and differentiation makers, inclusive of GATA4, NKX2.5, TBX5, MYH6, MYH7, and TNNI [108].

Another lncRNA *Fendrr* (Fetal-lethal non-coding developmental regulatory RNA), which is restricted to the caudal end of the lateral mesoderm, is located upstream of and co-expressed with *Foxf1*. *Fendrr* was found to occupy the promoters of specific transcriptional factor (*Foxf1, Pitx2 and Irx3*) by modulating PRC2 and TrxG/MLL complexes, leading to increased level of H3K27me3 and epigenetic silencing of these transcription factor genes. Moreover, knockdown of *Fendrr* in embryonic stem cells significantly increases the level of H3K4me3 at the promoter of *Gata6* and *Nkx2–5*, resulting in gene activation [109].

*PITX2* expression is a critical factor that determines left-right organ pattern and gut looping during development. Several lncRNAs have been reported to control *PITX2* expression. PANCR (PITX2 adjacent noncoding RNA) and *PITX2* express coordinately during the cardiogenic differentiation. Knockdown of *PANCR* decrease the expression of *PITX2* in hESC-derived cardiomyocytes, suggesting that *PITX2* expression require PANCR [110, 111]. Another lncRNA *Playrr* (Pitx2 locus-asymmetric regulated RNA), transcribed from right-specific enhancer element 926 (e926), is expressed on the right side and repressed by Pitx2 and *Playrr* and *Pitx2 were found to* exhibit mutual antagonism. These genes are parts of topologically associating domain (TAD). Conformational changes which alter the distance between the *Pitx2* and *Playrr* loci modulate their expression in this TAD, and CTCF (CCCTC-binding factor), a sequence-specific scaffold protein, is required to this interaction between *Pitx2 and Playrr* [112].

HAND2, a transcription factor playing a critical role in cardiac development and reprogramming cardiac fibroblasts into cardiomyocytes, was found to be regulated by lncRNAs *Upperhand and Handdown,* two lncRNAs that were transcribed from upstream and downstream of *Hand2* DNA locus, respectively. *Upperhand*, also named as *Uph* or *Hand2os1*, is a cardiac-enriched lncRNA that shares a bidirectional promoter with *Hand2*, has been shown to be essential for the transcription of *Hand2*. Anderson et al. revealed that *Uph* regulates the expression of *Hand2* through recruiting GATA4 to interact with *Uph-HAND2* super-enhancer. Knockout of lncRNA *Uph* locus resulted in congenital heart defects and perinatal lethality in mice, demonstrating the essential role of Uph-Hand2 regulatory partnership in cardiac development [113]. On the other hand, another lncRNA locus *Handdown* (also named as *Hdn*), located 7.2 kb downstream of *Hand2,* is active in early cardiac cells. The transcriptional activity of *Hdn* locus, but not *Hdn* transcript itself, suppresses Hand2 expression and is essential for murine development [113–115].

Tetralogy of Fallot (TOF) is one of the most common congenital heart diseases, characterized by defective ventricular septum and irregular cardiomyocyte differentiation, leading to the narrowing of right ventricular outflow tract (RVOT). Wang et al. reported that increased cardiac tissue (RVOT) levels of *HA117*, a lncRNA known to have anti-differentiation function, is associated with poorer McGoon ratio, Nakata index, and left ventricular end-diastolic volume index (LVEDVI) in patients with TOF [116]. In addition, patients with higher levels of *HA117* had longer cardiopulmonary bypass (CPB) time, ICU stay and poorer percentage improvement in SpO2 at 6 months after surgery. Although the functional contribution of *HA117 to the pathogenesis of* TOF remains unknown, Wang et al. proposed that *HA117* could serve as a prognostic biomarker for TOF [116]. Table 6 summarizes lncRNAs that are involved in the development of cardiovascular system.

**Table 6:** LncRNAs involved in cardiovascular development

LncRNA	Physiological/pathological impact	Mechanism involved
Bvht [106]	Bvht is essential for promoting ESC differentiation to cardiovascular cell fate	1. Upstream of MesP1 to promote MesP1-related gene expression 2. Modulating cardiovascular commitment through interacting with PRC2
Linc1405 [107]	Participate in developmental process, activate primitive streak differentiation.	1. Recruit Eomes to bind on *MesP1* promotor
CARMEN [108]	Inhibit the expression of Bvht to control the cardiac differentiation, mediate the cardiac transcriptional factors, play roles in cardiac specification and homeostasis	1. Upstream of Bvht to function with EZH2 and SUZ12
Fendrr [109]	Regulate histone modifier complexes, restrict the caudal end of the lateral mesoderm formation	1. Downstream modulate *Foxf1* 2. Occupy the *Foxf1, Pitx2 and Irx3* promotors to reduce the genes expression 1. Inhibit the promoter of *Gata6* and *Nkx2–5*
PANCR [110, 111]	Indirect but necessary to activate CM differentiation	1. Coordinately improve the cardiogenic differentiation with *PITX2C*
PLAYRR [112]		1. Act as *PITX2C* exhibit mutual antagonism
Upperhand (Uph) [113]	Reprogramming cardiac fibroblasts into cardiomyocytes	1. Upregulate the expression of HAND2
Handdown (Hdn) [113–115]	Reprogramming cardiac fibroblasts into cardiomyocytes	1. Interact with Uph to regulate HAND2 level
HA117 [116]	Anti-differentiation function in leukemia and Hirschsprung’s disease	1. Regulate the neighboring gene, *DPF3* and *RGS6* 2. Hypothesis as a biomarker for TOF

## LncRNAs in cardiac regeneration

LncRNA *CAREL* (cardiac regeneration-related lncRNA), a lncRNA that was continuously upregulated in the postnatal heart in parallel with the loss of regenerative capacity, was found to suppress cardiomyocyte division and proliferation by functioning as a ceRNA for miR-296. MiR-296 suppresses the expression of Trp53inp1, a protein known to repress the proliferation in neural stem cell [117], as well as Itm2a, a protein essential for chondrogenic cellular differentiation [118]. Low expression levels of *CAREL increases* miR-296, thereby repressing the levels of anti-proliferative Trp53inp1 and Itm2a proteins, leading to the regeneration and differentiation of cardiomyocytes both in vitro and in vivo. Myh6-driving cardiomyocyte-specific *CARE* trans-genic (TG) mice had lower cardiac regenerative capacity, which was rescued by miR-296 [119].

*CPR* (cardiomyocyte proliferation regulator) is a cardiac-restricted lncRNA that shows significant upregulation in adult than in embryonic mouse heart [120]. The percentage of EdU, pH 3 and Aurora B-positive CMs was significantly increased in the *CPR* KO neonatal mice, indicating the deletion of *CPR* promoted postnatal CM proliferation. By contrast, neonatal cardiomyocyte proliferation and cardiac regeneration were significantly suppressed in transgenic mice with cardiac-specific overexpression of *CPR* (*CPR* Tg). Although the gross morphology of *CPR* Tg mouse heart was practically identical to that in WT, the biomarkers of cardiac hypertrophy, including ANF (atrial natriuretic factor) and β-MHC (β myosin heavy chain) were substantially upregulated in *CPR* Tg, compared to WT control, mouse heart. These results suggest that overexpressing lncRNA *CPR* induce cardiac hypertrophic response instead of the CM proliferation in adult mice. Mechanistically, CPR was found to function by targeting minichromosome maintenance 3 (*Mcm3*), an initiator of DNA replication and cell cycle progression, thereby suppressing cardiomyocyte proliferation [120, 121]. *CPR* inhibits MCM3 transcription by directly interacting with and recruiting DNMT3A (DNA-methyltransferase 3A) at *Mcm3* promoter, leading to hypermethylation and suppressed *Mcm3* promoter activity [120].

NR_045363, a mice ortholog lncRNA of human LOC101927497, was remarkably conserved in other mammals and birds during neonatal cardiac regeneration. The overexpression of NR_045363 was found in the period of embryonic and neonatal stages and gradually reduced from childhood to adulthood. LncRNA NR_045363 promotes CM proliferation and cardiac regeneration by sponging miR-216a, a negative regulator of JAK2/STAT3 signaling pathway [122].

In another study, comparative RNA-Seq analyses on fetal and normal adult human heart identified lncRNA *NONHSAG007671*, or *CRRL* (cardiomyocyte regeneration-related lncRNA), as a novel mediator of CM regeneration. *CRRL* is highly conserved across species, including human, chimp, mouse and rat. Loss of *CRRL* significantly attenuated post-MI LV remodeling and fibrosis compared to control. Knockdown of *CRRL* in vivo enhanced cardiac function and activated CM proliferation, but not hypertrophy, after myocardial infarction. Mechanistically, CRRL acts as a ceRNA by sponging miR-199a-3p, thereby increasing the expression levels of *Hopx*, a miR-199a-3p target gene that plays a critical role in repressing CM proliferation [123]. Using a similar approach, the same group identified another CM proliferation regulator lncRNA *ECRAR* (Endogenous cardiac regeneration-associated regulator). Overexpression of *ECRAR* increased the number of EdU, pH 3, aurora B-positive cardiomyocytes in both postnatal and adult rat hearts after 14 days of the MI surgery, suggesting that *ECRAR* modulated CM proliferation and post-MI regeneration without causing hypertrophy. *ECRAR* promotes CM proliferation by direct binding with and increasing the phosphorylation of ERK1/2, a critical mediator of cell cycle progression. In addition, further analysis revealed that E2F1 transcriptionally upregulates *ECRAR*. Therefore, E2F1-ECRAR-ERK1/2 form a positive feedback loop to drive CM proliferation [124].

Insulin-like growth factor (IGF) signaling has been shown to be essential for CM proliferation and cardiac regeneration [125, 126], Li et al. discovered that *Sirt1* antisense lncRNA was highly upregulated in cardiomyocytes following IGF 1 treatment [127]. *Sirt1* antisense lncRNA is highly expressed in the embryonic day 16.5 (E16.5) CMs and sharply decreases from embryonic heart to postnatal heart. Overexpression of *Sirt1* antisense lncRNA promotes the proliferation of CMs, while depletion of *Sirt1* antisense lncRNA represses CM proliferation. Mechanistically, *Sirt1* antisense lncRNA stabilizes *Sirt1* mRNA by direct binding with the 3’UTR region of *Sirt1* mRNA [127]; moderately increased SIRT1 levels then promotes CM proliferation and cardiac regeneration.

*AZIN2-sv*, a splice variant of *AZIN2* (Antizyme inhibitor 2), is highly expressed in adult, compared to fetal, human heart [128]. Li et al. showed that *AZIN2-sv* repressed the proliferation of cardiomyocytes both in vitro and in vivo and the loss of *AZIN2-sv* stimulated the CMs proliferation and preserved cardiac function in post-MI mouse heart. Moreover, *AZIN2-sv* acts as a ceRNA for miR-214, a miRNA which negatively regulated the expression of PTEN (Phosphatase and tensin homolog). PTEN, a tumor suppressor that inhibits Akt/PKB signaling pathway, was also directly interacted with and stabilized by *AZIN2-sv*. Thus, the silence of *AZIN2-sv* promotes CM proliferation and cardiac regeneration through direct and indirect modulation of PTEN/Akt/PKB signaling pathway [128]. LncRNAs shown to be involved in the process of cardiac regeneration are summarized in Table 7.

**Table 7:** LncRNAs shown to be involved in the process of cardiac regeneration

LncRNA	Target	Physiological/pathological impact	Mechanism involved
CAREL [119]	MiR-296	Repress the cardiac regeneration and differentiation	A sponge for miR-296, repress miRNA-296, following by activate Trp53inp1 and Itm2a
CPR [120, 121]	DNMT3A	Induce cardiac hypertrophic response instead of the CM proliferation	Interact with DNMT3A to repress the level or MCM3
NR_045363 [122]	MiR-216a	Activate neonatal cardiac regeneration	Promote JAK2/STAT3 signaling pathway
CRRL [123]	MiR-199a-3p	Regulating CM proliferation	Protect Hopx from degeneration of CRRL
ECRAR [124]		Modulated CM proliferation and post-MI rehabilitation without causing hypertrophy	Promote the expression of cyclin D1, cyclin E1, and E2F1 proteins via ERK1/2 pathway
Sirt1 antisense lncRNA [127]	Sirt1 mRNA	Trigger the cardiac regeneration	Deacetylated and inhibited the activity of Nkx2.5, stabilized and increase the *Sirt1* mRNA expression
AZIN2-sv [128]	MiR-214	Stimulate CM proliferation	Increase the level of PTEN and inhibit Akt/PKB signaling pathway

## LncRNAs as biomarkers for cardiovascular diseases

The potential value of lncRNAs as diagnostic biomarkers has been widely explored. Although lncRNAs are not as abundant as other non-coding RNAs, the cell type- and disease-specific expression pattern make them suitable biomarker candidates. Because of the space limit, this review only covers a few of the important and well-established lncRNAs as biomarkers for CV diseases.

As mentioned earlier, ANRIL, located adjacent to the 9p21.3 risk locus, has been linked to increased CAD risk in several GWAS. This increased CAD risk associated with the single-nucleotide polymorphisms (SNPs) in this region is independent of all known CAD risk factors [93]. Interestingly, the risk alleles for atherosclerosis-related phenotypes were consistently associated with a low expression levels of ANRIL splice variant spanning exons 1–2, but not exon 17–18, of ANRIL, suggesting that different splicing variants of ANRIL might play distinct roles [129]. Indeed, different ANRIL splice variants have distinct expression patterns in peripheral blood mononuclear cells (PBMCs) from carriers of the risk haplotype, which suggests that differential splicing or transcript stability may confer different atherosclerosis susceptibility [130]. In one study enrolling 414 patients with acute myocardial infarction (AMI) treated by primary percutaneous coronary intervention, levels of hypoxia inducible factor 1A antisense RNA 2, KCNQ1OT1, MALAT1 and ANRIL in peripheral blood cells were significantly altered with AMI. Among them, ANRIL and KCNQ1OT1 improved the prediction of post-MI left ventricular dysfunction in a multivariate, prognostic regression model that includes demographic features, clinical parameters, and cardiac biomarkers [131].

CoroMarker, also named as Aldo-Keto Reductase Family 1 Member B1 Pseudogene 3, was discovered in a cohort study of patients receiving diagnostic coronary angiography for suspected CAD. CoroMarker from PBMCs was found to be a good biomarker with high sensitivity and specificity for the diagnosis of CAD. The expression levels of CoroMarker showed positive correlation with genes involved in atherosclerosis. Of note, CoroMarker was stable in plasma [132] and knockdown of CoroMarker decreased the production of pro-inflammatory cytokines from THP-1 monocytes [133]. However, the exact mechanisms via which CoroMarker regulates monocytes or atherosclerosis remain to be determined.

LIPCAR (long intergenic noncoding RNA predicting cardiac remodeling), a mitochondria-derived lncRNA, is highly expressed and consistently detectable in human plasma samples. Plasma LIPCAR has been shown to be an independent predictor for CAD and correlates with the severity of clinical presentation (highest in patients with AMI) [134]. In another study on patients with AMI, LIPCAR was downregulated early after AMI but upregulated during later stages, suggesting its role in chronic heart failure. Consistent with this observation, plasma LIPCAR level is elevated even more in CAD patients with heart failure. In addition, LIPCAR expression level is associated with future maladaptive cardiac remodeling in patients who experienced an episode of AMI. Of note, LIPCAR is independently associated with cardiovascular mortality in patients with chronic heart failure, regardless of the pathogenesis [135]. The mechanism, however, underlying the correlation between LIPCAR and CAD/AMI remains unclear.

LncRNA GAS5 was significantly increased in the plaque of atherosclerosis patients compared to normal people [136]. However, the expression level of plasma lncRNA GAS5 was significantly lower in patients with CAD. GAS5 decreased the level of p-mTOR without change of total mTOR in human coronary artery endothelial cells, which is an important initiator of pro-inflammatory response of monocytes/macrophages [137]. Furthermore, gain- and loss- of function studies showed that GAS5 modulates macrophages and ECs apoptosis in vitro. Interestingly, these effects of GAS5 on EC apoptosis is mediated by macrophage derived exomes after oxLDL stimulation, which demonstrated the interplay of macrophage and EC during atherosclerosis development [138].

SENCR (Smooth muscle and Endothelial cell enriched migration/differentiation-associated long NonCoding RNA) is highly expressed in ECs, SMCs and aortic tissues (vascular-enriched lncRNA) [139]. SENCR, localized mainly in the cytoplasm, stabilizes the contractile state of VSMCs by increasing myocardin expression [139]. Moreover, it was found that SENCR contributes to endothelial commitment in pluripotent cells and angiogenic capacity of ECs. SENCR expression was diminished in vascular ECs derived from superficial forearm veins of patients with critical limb ischemia and pre-mature coronary artery disease [140]. Using FISH-Flow assay, SENCR is downregulated in circulating ECs, but upregulated in monocytes, in early-onset CAD patients (EOCAD). Moreover, the combination of four molecular markers (intra-circulating EC SENCR, intra-monocyte SENCR, surface/intra-circulating EC CD146 and surface/intra-monocyte CD14) along with diabetes mellitus may serve as the early diagnostic tool for EOCAD [141].

Cardiovascular (CV) diseases are the major cause of morbidity and mortality in patients with end-stage renal disease (ESRD), accounting for nearly 50% of deaths in this population [142, 143]. In a cohort of patients with chronic kidney disease, end-stage renal disease (ESRD) with or without cardiovascular (CV) event, circulating lncRNA expression profiles discriminate between ESRD patients with and without an adverse CV event. Among the differentially expressed lncRNAs, eight plasma lncRNAs were identified as potential predictors of adverse CV outcomes in uremic patients, and lncRNA DKFZP434I0714 was confirmed as an independent predictor of adverse CV outcomes in patients with ESRD. LncRNA DKFZP434I0714 is not dysregulated in human failing heart, but it is shown to regulate endothelial function. Gain- and loss- of function studies showed lncRNA DKFZP434I0714 modulates stress-induced EC apoptosis, endothelial dysfunction, and vascular inflammation, which are hallmarks of vascular complications associated with uremia [144]. Table 8 summarizes the examples of lncRNAs that have been shown to be potential biomarkers for cardiovascular diseases.

**Table 8:** LncRNAs as biomarkers for cardiovascular diseases

LncRNA	Clinical Application	Physiological/pathological impact	Mechanism involved
CoroMarker [132, 133]	Diagnosis of CAD	Decrease pro-inflammatory cytokine secretion from THP-1 monocytic cells	Unknown
LIPCAR [135]	Prediction of cardiac remodeling	Associated with future development of cardiac remodeling	Unknown
GAS5 [136–138]	↑ in arterial plaques ↓ in plasma of CAD patients	Modulate macrophages and ECs apoptosis after ox-LDL stimulation	Unknown
SENCR [139–141]	Diagnosis of early onset CAD: ↓ in circulating ECs ↑ in monocytes	Regulation of commitment from pluripotent cells and angiogenic capacity of EC	Regulate myocardin gene regulation to stabilize the contractile state of VSMCs
DKFZP434I0714 [144]	Prediction of adverse CV events in uremic patients	Modulate stress-induced EC apoptosis, endothelial dysfunction, and vascular inflammation	Unknown

Even though the cellular and pathological specificity of lncRNAs make them as suitable biomarkers, using lncRNAs as clinical biomarkers is potentially limited by the difficulties in their isolation and quantification. RNA is very difficult to isolate in reasonable quantities from acellular bodily fluids such as plasma or serum. In addition, the high cost and low throughput associated with RNA processing and quantification also limits the application of lncRNA as a biomarker. For example, it seems unlikely that lncRNA could replace cardiac troponins in the diagnosis of AMI, as clinical tests for cardiac troponins are relatively cheap, fast and well-validated. Therefore, the potential usage of lncRNAs as biomarkers is more likely to be prognostic, rather than diagnostic, in cardiovascular diseases.

## Conclusions

Emerging evidence indicates the important roles of lncRNAs in the complex regulatory network of cardiovascular development and diseases. It has been well-demonstrated that many of these lncRNAs could be utilized as novel therapeutic targets and/or biomarkers for diagnosis/prognosis for cardiovascular diseases including cardiac hypertrophy, myocardial infarction, heart failure and atherosclerosis. It will require extensive efforts, however, to refine the approaches of modulating lncRNA expression in vivo and to improve/standardize the quantitative assays for lncRNA biomarkers to make clinical translation possible.

## Data Availability

Not applicable.
